# Draft Genome Sequence of Trebouxiophyceae sp. Strain KSI-1, Isolated from an Island Hot Spring

**DOI:** 10.1128/MRA.01185-18

**Published:** 2018-10-25

**Authors:** Hiro Takahashi, Satoshi Tanaka, Shuhei Hayashi, Shido Miyaki, Anna Takahashi, Shinnosuke Onai, Shuichi Fukuyoshi, Hitoshi Miyasaka

**Affiliations:** a Graduate School of Medical Sciences, Kanazawa University, Kanazawa, Ishikawa, Japan; b Graduate School of Horticulture, Chiba University, Matsudo, Chiba, Japan; c Fundamental Innovative Oncology Core Center, National Cancer Center, Chuo-ku, Tokyo, Japan; d Advanced Technology Laboratory, Kansai Electric Power Co., Sourakugun, Kyoto, Japan; e Department of Applied Life Science, Sojo University, Nishiku, Kumamoto, Japan; f Faculty of Information Technologies and Control, Belarusian State University of Informatics and Radio Electronics, Minsk, Belarus; g Institute for Theoretical Physics, Kanazawa University, Kanazawa, Ishikawa, Japan; h Institute of Medical, Pharmaceutical, and Health Sciences, Kanazawa University, Kanazawa, Ishikawa, Japan; Indiana University Bloomington

## Abstract

Trebouxiophyceae sp. KSI-1 is a green alga isolated from a seashore hot spring on Satsuma Iōjima in Kagoshima, Japan, and is highly tolerant to oxidative stress. Here, we report the draft genome sequence of this strain, thereby providing an insight into the genetic basis for its oxidative stress tolerance.

## Abstract (precis)

Trebouxiophyceae sp. KSI-1 is a green alga isolated from a seashore hot spring on Satsuma Iōjima in Kagoshima, Japan, and is highly tolerant to oxidative stress.

## ANNOUNCEMENT

Reactive oxygen species (ROS) are generated by electron leakage to O_2_ in the electron transport system. In oxygen-evolving photosynthetic organisms, various stresses accelerate the generation of ROS generation (1) due to disruption in the balance between photosynthetic excitation energy and terminal energy consumption for CO_2_.

Trebouxiophyceae sp. strain KSI-1 was isolated from a seashore hot spring on Satsuma Iōjima, a volcanic island in Kagoshima, Japan. This strain was grouped in a new genus within the class Trebouxiophyceae based on its 18S rRNA sequence (GenBank accession number LC082307).

Modified Okamoto medium (MOM) (2), containing 3% NaCl, was used for the algal cultures. For the isolation of oxidative stress-tolerant green algae, the aqueous sample was mixed with an equal volume of MOM (pH 8.0) and cultured under continuous illumination at a light intensity of 100 μmol/m^2^/s of photosynthetically active radiation (PAR), with aeration by bubbling at a rate of 200 ml air/min. After the growth of green algal cells was observed, enrichment culture for the isolation of oxidative stress-tolerant green algae was initiated by culturing the cells under a stressed condition of 50 μM methyl viologen (MV). Passage cultures were repeated three times under the stressed condition, and finally, the stress-tolerant green algal cells were purified from the monoculture by streaking the cells onto the MOM agar plates and isolating the algal colony. Genomic DNA was isolated from the cells (ca. 50 mg) using a DNeasy plant minikit (Qiagen) according to the manufacturer’s instructions after being disrupted in liquid nitrogen using a mortar and pestle. A 500-ng aliquot of DNA was sonicated to generate an average of 600-bp DNA fragments for library preparation. End repair, A tailing, adaptor ligation, PCR, and library purification were performed as described in our previous study (3). The sequencing of a TruSeq DNA library (paired-end 2 × 300-bp reads) generated 33,531,306 reads. Removal of the sequencing primers and trimming of the low-quality read regions from the obtained short reads were conducted with the CLC Genomics Workbench version 11.0.1 (Qiagen) with default parameters. *De novo* assembly was also conducted with the CLC Genomics Workbench. The resulting genome assembly had a length of 44,347,176 bp divided into 22,485 contigs; the *N*_50_ contig length was 21,380 bp, the GC content was 64.6%, and genome coverage was 194.9×.

MV causes severe oxidative stress (4). Trebouxiophyceae sp. KSI-1 shows a surprisingly high tolerance to oxidative stress caused by MV and copper ions. Higher plants can generally tolerate MV and copper ions at concentrations up to 5 and 100 μM, respectively, while Trebouxiophyceae sp. KSI-1 was tolerant to concentrations of 100 and 10 mM, respectively. Trebouxiophyceae sp. KSI-1 is, therefore, a prominent genetic resource of unique antistress genes for the molecular breeding of stress-tolerant plants (5), and the genomic information of this alga may provide insight into the genetic basis for oxidative stress tolerance in photosynthetic organisms.

### Data availability.

The draft genome sequences for Trebouxiophyceae sp. KSI-1 have been deposited in DDBJ/ENA/GenBank under accession numbers BHFV01000001 to BHFV01022485. The SRA/DRA/ERA accession number is DRA007300.
